# B Chromosomes in Grasshoppers: Different Origins and Pathways to the Modern B_s_

**DOI:** 10.3390/genes9100509

**Published:** 2018-10-18

**Authors:** Ilyas Yerkinovich Jetybayev, Alexander Gennadievich Bugrov, Victoria Vladimirovna Dzyubenko, Nikolay Borisovich Rubtsov

**Affiliations:** 1 The Federal Research Center Institute of Cytology and Genetics, Russian Academy of Sciences, Siberian Branch, Lavrentjev Ave., 10, 630090 Novosibirsk, Russia; rubt@bionet.nsc.ru; 2 Institute of Systematics and Ecology of Animals, Russian Academy of Sciences, Siberian Branch, Frunze str. 11, 630091 Novosibirsk, Russia; bugrov@fen.nsu.ru; 3 Novosibirsk State University, Pirogov str., 2, 630090 Novosibirsk, Russia; victoriad@mail.ru

**Keywords:** B chromosomes, grasshoppers, DNA composition, repeat clusters, euchromatin degradation, microdissected DNA probes

## Abstract

B chromosomes (B_s_) were described in most taxa of eukaryotes and in around 11.9% of studied Orthopteran species. In some grasshopper species, their evolution has led to many B chromosome morphotypes. We studied the B_s_ in nine species (*Nocaracris tardus, Nocaracris cyanipes, Aeropus sibiricus, Chorthippus jacobsoni, Chorthippus apricarius, Bryodema gebleri, Asiotmethis heptapotamicus songoricus, Podisma sapporensis,* and *Eyprepocnemis plorans*), analyzing their possible origin and further development. The studied B_s_ consisted of C-positive or C-positive and C-negative regions. Analyzing new data and considering current hypotheses, we suggest that B_s_ in grasshoppers could arise through different mechanisms and from different chromosomes of the main set. We gave our special attention to the B_s_ with C-negative regions and suggest a new hypothesis of B chromosome formation from large or medium autosomes. This hypothesis includes dissemination of repetitive sequences and development of intercalary heterochromatic blocks in euchromatic chromosome arm followed by deletion of euchromatic regions located between them. The hypothesis is based on the findings of the *Eyprepocnemis plorans* specimens with autosome containing numerous intercalary repeat clusters, analysis of C-positive B_s_ in *Eyprepocnemis plorans* and *Podisma sapporensis* containing intercalary and terminal C-negative regions, and development of heterochromatic neo-Y chromosome in some Pamphagidae grasshoppers.

## 1. Introduction

The B chromosomes (B_s_) were initially described in 1907 as additional elements to the standard karyotype in species of the *Metapodius* genus (Hemiptera) [1]. The number of eukaryotic species with B_s_ were estimated from 1685 [2] in 1980 to more than 2828 [3] in 2017. Among Orthopteran species, B_s_ were described in 191 species, which constitutes 11,9% of studied species [4]. Often B_s_ are enriched with repetitive sequences and are highly heterochromatic. The presence of B_s_ is non-essential for organism development and provides little or no effect [5,6,7,8].

Despite intensive studies and wide range of used methods, the origin and further evolution of B_s_ remains an intriguing question. None of the approaches can provide compelling evidence on this topic. Modern views on these questions are still full of hypotheses and assumptions. However, all researchers agree, that the B_s_ can arise either from rearranged chromosomes of a basic set or from material resulted from interspecific hybridization [5].

In the former case, the B_s_ often derive from pericentric regions of A chromosomes (A_s_) which resulted from deletion of the whole or almost of the whole euchromatic part of a chromosome. The B_s_ originated from this event are small and mostly C-positive. However, there are many other morphotypes of B_s_ in grasshoppers, where the mechanism of their origination and further evolution is not so clear.

Frequently the X chromosome and smallest autosomes are suggested as the possible ancestors of the B_s_ in grasshoppers: the X shows special meiotic behavior and dosage compensation mechanisms that can reduce problems caused by partial aneuploidy [5,6,9,10]. Small autosomes traditionally were also considered as a possible ancestor of the B_s_ because they contain fewer genes and thus additional small autosome could be better tolerated than other large A_s_ [9]. Recently it was shown that some small autosomes usually contain many repetitive DNA [11,12,13] strands and often they contain large additional C-positive blocks on their distal part [14,15].

Because of heteropicnosity and univalency of many B_s_, they resemble the X in meiosis. This similarity is a circumstantial evidence in favor of the X chromosome origin of B_s_ but it cannot be acknowledged to be the direct proof [10]. Molecular markers also do not provide compelling evidence of the B chromosome origin. For instance, mutual location of 180 bp satellite DNA (satDNA) and ribosomal DNA (rDNA) cluster on the B_s_ and on the X in *Eyprepocnemis plorans* supported the hypothesis that the X was the ancestor of these B_s_ [16,17]. However, sequencing of internal transcribed spacer (ITS) of 45S rRNA gene revealed unique variants of the ITS present in each chromosome. The ITS from the autosome S11 appeared to be the closest one to the ITS from the B chromosome [18]. We should also note that sequencing of B_s_ in six mammalian species showed that B_s_ can contain DNA fragments from different A_s_ [19,20,21], which significantly puzzles the question of the B_s_ origin.

The large autosomes are rarely considered as possible ancestors of the B_s_ [22]. Furthermore, most of the B chromosome studies are focused on C-positive B_s_ and rarely discuss the origin C-negative B chromosome regions. Here, we described B_s_ in 9 grasshopper species, examined their various origins including the large or medium autosomes, and proposed that they also might be considered as an ancestor of such C-negative B_s_.

## 2. Material and Methods

Specimens of nine grasshopper species were collected during field season of 1987 and 2000–2017 in different locations indicated in the Table 1.

### 2.1. Chromosome Preparation

Testes of males were fixed in the field according to standard methods [23]. Females were kept alive and reared in our laboratory to obtain eggpods. The meiotic chromosomes and metaphase chromosomes of somatic cells were prepared from male testes and embryos according to standard procedures [24].

### 2.2. DNA Probes Generation

Chromosome microdissection was performed according to standard procedure as described earlier [24]. Microdissected DNA libraries (Table 2) were generated according to standard protocol from 8–28 copies of the chromosome or chromosome region collected by extended glass needle using a micromanipulator MR (Zeiss, Oberkochen, Germany). The WCPNtaB (Whole Chromosome Paint of *Nocaracris tardus* B) DNA probe was generated from 20 copies of B_s_ of the *Nocaracris tardus* specimen with the 4 B_s_ per cell. The PCPCapBq (Partial Chromosome Paint of *Chorthippus apricarius* B q arm) DNA probe was generated from the 8 copies of long arm of the B chromosome and PCPCapAc (Partial Chromosome Paint of *Chorthippus apricarius* A centromeric region)) DNA probe was generated from 8 copies of pericentromeric C-positive region of one of autosome of *Chorthippus apricarius*. A microdissected WCPEplBa4 (Whole Chromosome Paint of *Eyprepocnemis plorans* Ba4) DNA probe was generated from eight copies of the Ba4 of *Eyprepocnemis plorans*. The amplification of DNA isolated from the microdissected material was carried out using polymerase chain reaction (PCR) with partly degenerated primer MW6 (DOP-PCR) [25] as described earlier [26]. The PCPPsaB1-B2dist (Partial Chromosome Paint of *Podisma sapporensis* distal parts of B1 and B2) DNA probe was generated from 28 copies of distal C-negative regions of the B1 and B2 chromosomes of *Podisma sappoernsis*. Dissection was performed from central part of B1-B2 bivalent that formed chiasma in the distal parts of the B_s_. Dissected material was amplified using a GenomePlex Single Cell Whole Genome Amplification Kit (WGA4) (Sigma-Aldrich, St. Louis, MO, USA) according to the manufacturer’s protocol. DNA labelling was carried out in additional 15 cycles of PCR in presence of Flu- or TAMRA-dUTP (Genetyx, Novosibirsk, Russia) in additional 20 PCR cycles using WGA3 kit (Sigma-Aldrich, St. Louis, MO, USA) or with 20 high temperature cycles of DOP-PCR. DNA probe for rDNA and telomeric repeats was prepared as previously described [27].

### 2.3. C-Banding and Fluorescence In Situ Hybridization (FISH) Technique

C-banding and fluorescence in situ hybridization (FISH) were performed as described earlier [28,29].

### 2.4. Microscopy

Microscopy was performed at the Centre for Microscopy of Biological Objects (Institute of Cytology and Genetics SB RAS, Novosibirsk, Russia) with an AxioImager.M1 (Zeiss) fluorescence microscope equipped with #49, #46HE, #43HE filter sets (Zeiss), ProgRes MF CCD camera (JenaOptik, Jena, Germany), using Software package ISIS5 (MetaSystems GmbH, Altlussheim, Germany).

## 3. Results

### B Chromosome Morphology in Studied Grasshopper Species

We analyzed B_s_ in nine species of grasshoppers: *Nocaracris tardus*, *Nocaracris cyanipes*, *Aeropus sibiricus*, *Chorthippus jacobsoni*, *Chorthippus apricarius*, *Bryodema gebleri, Asiotmethis heptapotamicus songoricus*, *Podisma sapporensis*, and *Eyprepocnemis plorans*.

**Figure 1:**
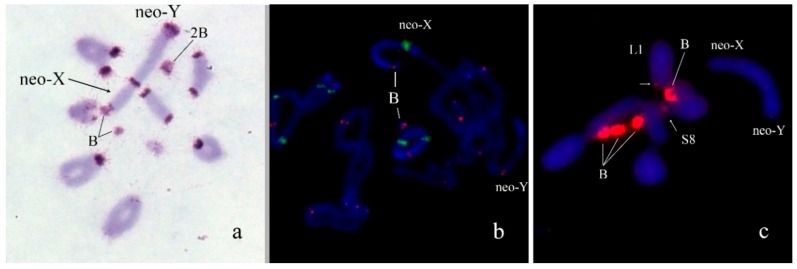
B_s_ in meiotic cells of *Nocaracris tardus: (***a**) C-banding; (**b**) fluorescence in situ hybridization (FISH) with ribosomal DNA (rDNA) (green) and telomeric DNA (red) probes; (**c**) FISH with WCPPtaB (red) DNA probe. Arrows indicate localization of hybridization signals in pericentric regions of L1 and S8 autosomes. Chromosomes counterstained with 4′,6-Diamidino-2-Phenylindole, Dihydrochloride (DAPI) (blue).

In *Nocaracris tardus* (2n = 18 + neo-XX♀/neo-XY♂) B_s_ were found in 6 out of 7 karyotyped specimens. Number of the B_s_ varied in different specimens from 0 to 4 per cell. All revealed B_s_ were C-positive and very small. Their size was approximately equal to C-positive blocks detected in the most of autosomes (Figure 1a). On early stages of meiosis, these B_s_ conjugated with the terminal region of the short arm of the neo-X chromosome (Figure 1b). Two-color FISH with labelled rDNA and telomeric DNA probe showed only telomeric repeats on the B chromosome termini (Figure 1b). FISH with WCPNtaB revealed strong hybridization signal only on the B_s_. We observed weaker hybridization signal in centromeric regions of L1 and S8 autosomes (Figure 1c).

In *Nocaracris cyanipes* (2n = 18 + neo-XX♀/neo-XY♂), the only B chromosome was found in the male from Elbrus region of Russia. It was the same as the B_s_ of *N. tardus* in size and C-banding pattern. Two-color FISH with labelled rDNA and telomeric DNA probe also showed only telomeric repeats clusters on the B chromosome termini. In meiosis, this B chromosome was often located close to a terminal region of the short arm of the neo-X chromosome.

**Figure 2:**
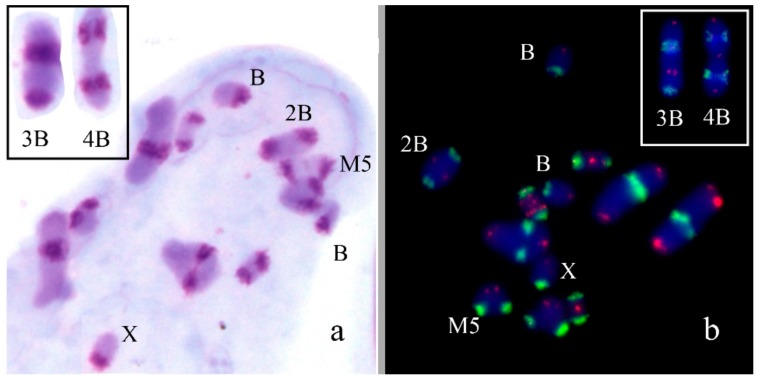
B_s_ in meiotic cells of *Aeropus sibiricus*: *(***a**) C-banding, inset shows trivalent and tetravalent of B_s_; (**b**) FISH with rDNA (green) and telomeric DNA (red) probes, chromosomes counterstained with DAPI (blue), inset shows trivalent and tetravalent of B_s_.

In *Aeropus sibiricus* (2n = 16 + XX♀/X0♂), four B_s_ were found in one male out of six analyzed specimens. All four B_s_ were present in all studied cells. All of them were medium-sized acrocentrics with pericentromeric C-positive region similar in size to pericentromeric C-positive regions of A_s_. They contain also large C-negative regions. In meiosis, they can be univalents or formed bi-, tri-, and tetravalent (Figure 2a). Two-color FISH with labelled rDNA and telomeric DNA probe revealed on the B_s_ rDNA in C-positive pericentric region and telomeric repeat clusters on the termini of B_s_ (Figure 2b). We observed the same distribution of these repeats in all chromosomes of the set.

**Figure 3:**
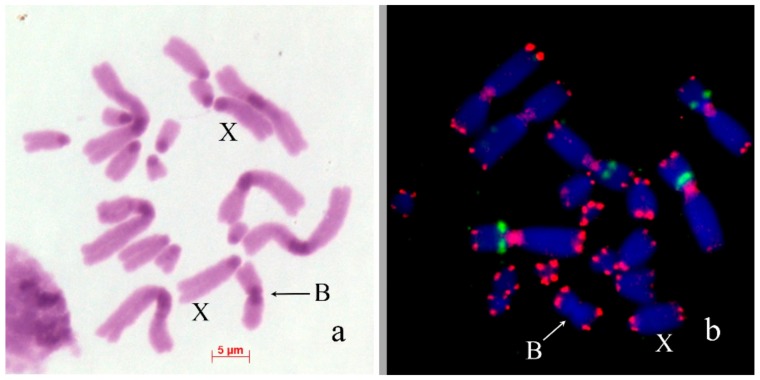
B_s_ in mitotic cells of *Chorthippus jacobsoni*: *(***a**) C-banding; (**b**) FISH with rDNA (green) and telomeric DNA (red) probes, chromosomes counterstained with DAPI (blue). Arrows indicate B_s_.

In *Chorthippus jacobsoni* (2n = 16 + XX♀/X0♂), the B_s_ were found in 15 out of 108 analyzed embryos, with one B chromosome per cell. They were metacentrics equal to the X in size with arms similar to arms of M6 autosome. They have large pericentromeric C-positive block and C-negative arms (Figure 3a). They looked like typical isochromosomes. Two-color FISH with rDNA and telomeric DNA probes showed telomeric clusters on the ends of the B_s_, the interstitial site of rDNA in two large metacentrics (Figure 3b). We did not observe any interstitial telomeric signals in pericentric C-positive block of B chromosome, in contrast to pericentric C-positive regions of large autosomes that contained the large cluster of these repeats.

**Figure 4:**
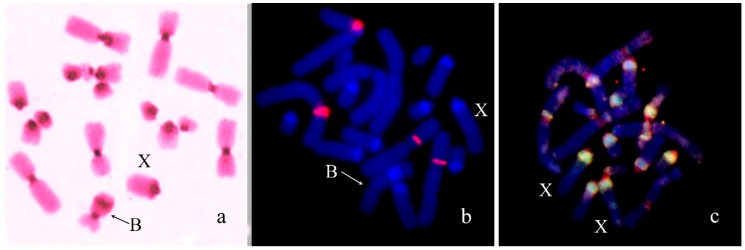
B_s_ in mitotic cells of *Chorthippus apricarius*: (**a**) C-banding; (**b**) FISH with rDNA (red) probe; (**c**) FISH of PCPCapBq (green) and PCPCapAc (red) with main chromosome set. Chromosomes counterstained with DAPI (blue). Arrows indicate B_s_.

In *Chorthippus apricarius* (2n = 16 + XX♀/X0♂), B_s_ were found in seven out of 52 analyzed embryos, with one B chromosome per cell. The B_s_ were submetacentrics equal to the X in size. C-banding revealed medium-sized pericentric C-positive block and large interstitial C-positive block in the long arm of the B_s_ (Figure 4a). FISH with labeled rDNA did not reveal clusters of rDNA in the B_s_ (Figure 4b). PCPCapBq intensively painted interstitial C-band in the B chromosome arm and distal parts of pericentric regions of most of A_s_. PCPCapAc painted all pericentric C-blocks (Figure 4c). In biarmed L1–L3 chromosomes, hybridization signals were significantly weaker and were observed only in distal part of the pericentric C-bands in their short arms. In L3 autosome, hybridization signals were weaker than in the L1 and L2 autosomes (Figure 4c).

**Figure 5:**
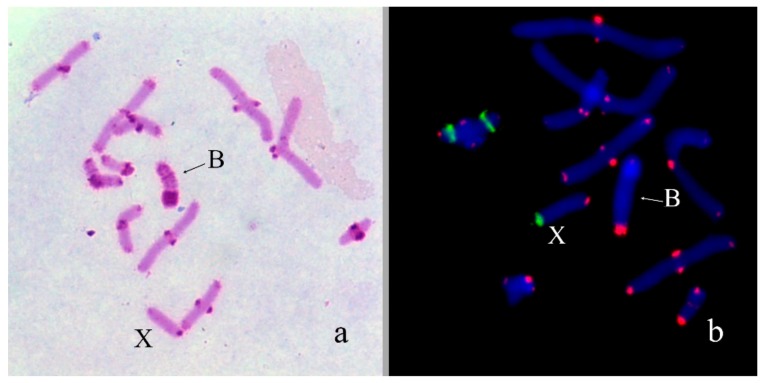
B_s_ in meiotic cells of *Bryodema gebleri*: (**a**) C-banding; (**b**) FISH with rDNA (green) and telomeric DNA (red) probes, chromosomes counterstained with DAPI (blue). Arrows indicate B_s_.

In *Bryodema gebleri* (2n = 22 + XX♀/X0♂), the only B was found in one out of 12 males from Kurai steppe in Altay region of Russia. It was large acrocentric chromosome with very small C-negative short arm, equal to L3 in size. The B chromosome contained small pericentromeric and six intercalary C-positive blocks in the long arm. Terminal large C-positive block was approximately 1/3rd of the B chromosome size (Figure 5a). Two-color FISH with labelled rDNA and telomeric DNA probe showed small telomeric repeat cluster on the end of long arm and a large cluster of telomeric repeats in short arm of the B. (Figure 5b).

**Figure 6:**
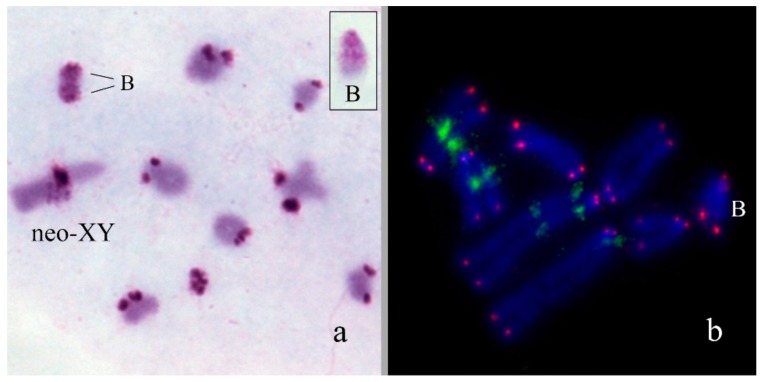
B_s_ in meiotic and mitotic cells of *Asiotmethis heptapotamicus songoricus*: (**a**) C-banding, inset shows mitotic B chromosome; (**b**) FISH with rDNA (green) and telomeric DNA (red) probes, chromosomes counterstained with DAPI (blue).

In *Asiotmethis heptapotamicus songoricus* (2n = 18 + neo-XX♀/neo-XY♂), the B_s_ were found in three out of 20 males from a population near the Ayaguz river, eastern Kazakhstan. In one specimen, two B chromosomes per cell were observed. The B_s_ were approximately equal to M6-S7 autosomes in size. They were partly C-negative and a proximal half of the B_s_ contained two interstitial C-positive blocks in addition to pericentromeric C-positive region. In meiosis of one specimen, two B_s_ formed bivalents (Figure 6a). Two-color FISH with labelled rDNA and a telomeric DNA probe showed only telomeric repeat clusters on the termini of the B_s_ (Figure 6b).

**Figure 7:**
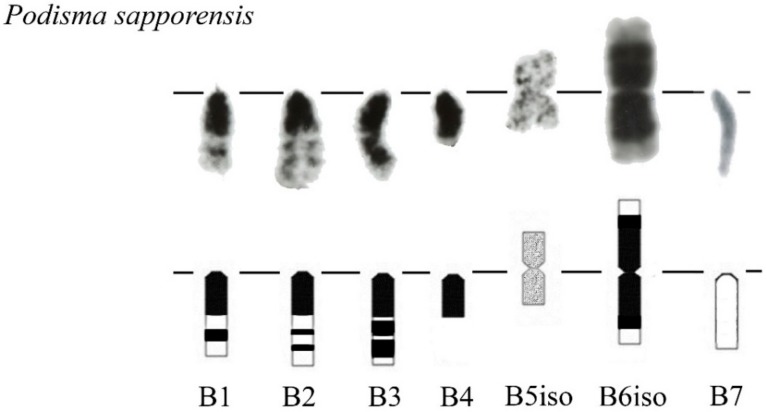
Morphotypes of B chromosome in *Podisma sapporensis* according to [30].

**Figure 8:**
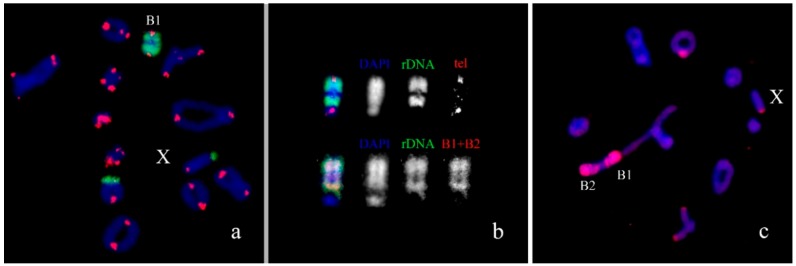
Fluorescence in situ hybridization (FISH) of DNA probes with meiotic cells of *Podisma sapporensis*: (**a**) rDNA (green) and telomeric DNA (red) probes; (**b**) localization of DNA probes on B1 chromosome; (**c**) PCPPsaB1-B2dist (red). Chromosomes counterstained with DAPI (blue).

We previously analyzed the populations of *Podisma sapporensis* (2n = 22 + XX♀/X0♂ and 2n = 20 + neo-XX♀/neo-XY♂) and described seven morphotypes of the B_s_ [24,30,31]. The B1, B2, B3, B4 and B6iso morphotypes correspond to heavily heterochromatic B_s_ with C-negative region(s) in the distal part of the chromosomes. In B1 and B2 morphotypes, interstitial C-negative regions were interlaced with C-positive ones. In the B3 morphotypes C-negative regions were small. The B6iso morphotype was characterized with terminal C-negative regions in both chromosome arms. The B5iso exhibited multiple small C-positive regions dispersed along the whole chromosome. Finally, the B7 chromosome was almost fully C-negative (Figure 7). In meiosis, the B1 and B2 conjugated with their distal C-negative regions (Figure 8c).

Fluorescence in situ hybridization (FISH) with rDNA and telomeric DNA probes showed that C-positive regions of B1 and B2 morphotypes were enriched with rDNA. We observed telomeric repeats on the ends of all chromosomes. In addition to these clusters, an interstitial telomeric cluster was revealed, on the border of their distal interstitial C-positive block and interstitial C-negative region in the B1 (Figure 8a,b). The PCPPsaB1-B2dist DNA probe was probably contaminated with material of C-positive regions. Consequently, FISH with PCPPsaB1-B2dist gave a very strong signal on C-positive regions of the B_s_, masking the signal in original C-negative region (Figure 8c). Patterns of FISH signal with this microdissected DNA probe and labeled rDNA on the B_s_ were very similar, suggesting that FISH with this microdissected DNA probe showed mainly the regions highly enriched with rDNA (Figure 8b).

**Figure 9:**
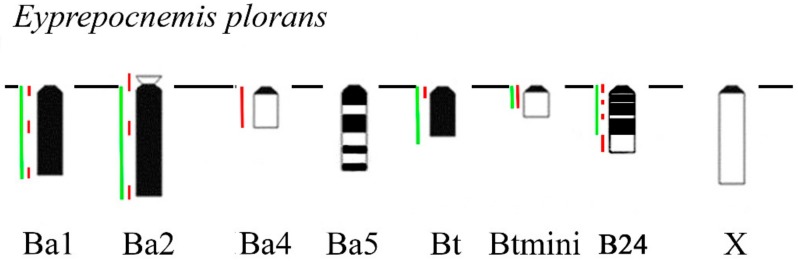
Schematic image of C-banding patterns and localization of rDNA (**green line**) and WCPEplBa4 DNA probes (**red line**) of different morphotypes of B_s_ in *Eyprepocnemis plorans*. The X is shown for size comparison.

**Figure 10:**
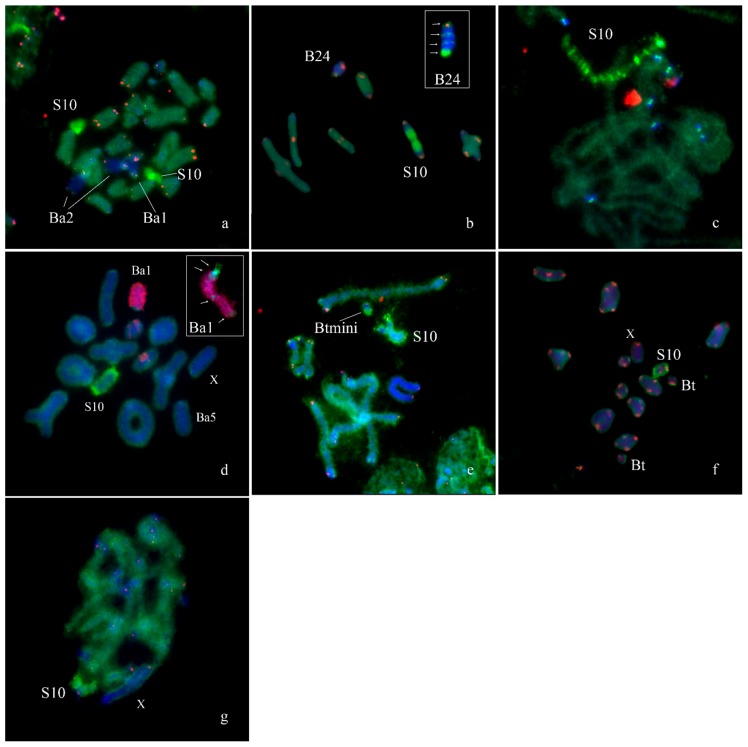
Fluorescence in situ hybridization of DNA probes with chromosomes of *Eyprepocnemis plorans:* (**a**) WCPEplBa4 (green) and telomeric DNA probe (red) with individual from Armenian population containing Ba1 and Ba2; (**b**) WCPEplBa4 (green) and telomeric DNA probe (red) with individual from Spanish population containing B24 (inset shows mitotic B24, arrows indicate signals from WCPEplBa4); (**c**) WCPEplBa4 (green) and rDNA probe (red) with pachytene chromosomes of individual from Armenian population; (**d**) WCPEplBa4 (green) and rDNA probe (red) with individual from Armenian population containing Ba1 and Ba5, inset shows mitotic Ba1, arrows indicate signals from WCPEplBa4; (**e**) WCPEplBa4 (green) and telomeric DNA probe (red) with individual from Turkish population containing Btmini; (**f**) WCPEplBa4 (green) and telomeric DNA probe (red) with individual from Turkish population containing BT chromosome; (**g**) WCPEplBa4 (green) and telomeric DNA probe (red) with individual of *Eyprepocnemis* *plorans meridionalis* from South Africa.

In *Eyprepocnemis plorans* (2n = 22 + XX♀/X0♂), five morphotypes of the B_s_ were revealed in eastern populations from Armenia and Turkey (Figure 9) [32]. The Ba1 and Ba2 were large acrocentrics, equal or larger to the X and mostly C-positive. Small C-negative arm was revealed in Ba2 chromosome. The Ba3 chromosome was fully C-negative, as well as Ba3iso. The Ba4 was equal in size to the smallest autosome and was mostly C-negative with small pericentric C-positive region [32]. The Ba5 was medium-sized with multiple interstitial C-positive blocks along the whole chromosome (Figure 9). Two additional morphotypes of the B_s_ were found in populations from Turkey [32]. The Bt chromosome was small C-positive acrocentric. The Btmini chromosome was smaller than Bt and C-negative (Figure 9). In addition to eastern morphotypes of B_s_, we studied B24 morphotype from Spain. It is a C-positive medium sized B chromosome with interstitial and distal C-negative regions. Regions of these B_s_ painted with labeled rDNA and WCPEplBa4 DNA probe are shown in Figure 9 and Figure 10.

Fluorescence in situ hybridization with WCPEplBa4 also intensively painted the whole S10 autosome on metaphase plates of specimens from Armenian (Figure 10a,c,d), Spanish (Figure 10b), Turkish (Figure 10e,f) and South African (Figure 10g) populations. In the Spanish population the S10 carried a large C-positive pericentric region that was not painted with the WCPEplBa4 while its C-negative part was strongly painted with the WCPEplBa4 (Figure 10b).

FISH with WCPEplBa4 also gave a dot-like signal in centromeric regions of all other A chromosomes of Armenian (Figure 10c) specimens. C-negative material of other chromosomes exhibited a weaker hybridization signal after FISH with WCPEplBa4.

We revealed unexpected painting pattern after FISH with the WCPEplBa4 and labeled rDNA on the Ba1. FISH with WCPEplBa4 gave a signal on the pericentromeric region, a weaker signal on the distal region of the long arm and a dot-like signal in the proximal region and in the middle of the long arm. At the same time, labeled rDNA painted the whole Ba1 but a proximal part of the pericentromeric region (Figure 10d). On the extended meiotic chromosomes, a hybridization signal of the WCPEplBa4 on S10 autosome was split into multiple intensive signals interlaced with weaker labeled regions (Figure 10c). The same FISH pattern was observed on the S10 of all studied individuals from Armenian, Turkish, Spanish and South African populations. A FISH pattern of the Ba2 obtained with WCPEplBa4 and labeled rDNA was similar to the pattern of the Ba1 (Figure 10a). The only distinct difference of the Ba2 was associated with its short arm that was painted with the WCPEplBa4. The FISH pattern of the Ba5 after FISH with WCPEplBa4 was similar to the FISH patterns of the A_s_ (Figure 10d). FISH with the WCPEplBa4 on the B_s_ from Turkish populations gave hybridization signal in distal part of the Btmini and in pericentric region of the Bt (Figure 10e,f). The FISH of WCPEplBa4 with the B24 from Spanish populations exhibited two interstitial hybridization signals in C-negative regions in 1/3rd and 2/3rd of the length and one in distal terminal region (Figure 10b).

## 4. Discussion

### 4.1. Origin of Dot-Like B_s_

The B_s_ in grasshoppers vary in size from minute dot-like elements to a large chromosome equal to the largest autosome (Figure 1, Figure 2, Figure 3, Figure 4, Figure 5, Figure 6, Figure 7, Figure 8, Figure 9 and Figure 10). We observed the dot-like B_s_ in *N. tardus* and *N. cyanipes* (Figure 1). The mechanism of chromosome rearrangement proving these dot-like B_s_ remains unknown; however, these chromosomes resemble human small supernumerary marker chromosomes (SMCs). Many small SMCs are dot-like chromosomes and usually they exhibit mitotic and meiotic instability leading to mosaicism of small SMCs in tissues [33]. The B_s_ of *N. tardus* also showed mosaicism. Two hypotheses were previously suggested for the mechanism of human small SMC origin: SMCs arise as a result of deletion of almost the whole arm(s) or by forming the inverted duplication of pericentromeric region of acrocentrics followed by inactivation of one of the centromeres [33]. In our study, the results of the FISH of WCPNtaB probe with metaphase chromosomes of *N. tardus* supports the hypothesis of their origination from a pericentromeric region of the acrocentrics. The DNA of the B chromosome showed homology to the pericentromeric regions of the L1 and S8 autosomes (Figure 1c) indicating them as the possible B chromosome ancestor. However, taking in account rapid evolution of repetitive DNA in the pericentromeric regions, we cannot regard these repeats as reliable phylogenetic markers.

The special features of *N. tardus* and *N. cyanipes* karyotype made us look for another hypothesis of the B chromosome origin in these species. In contrast to many grasshopper species, *N. tardus* and *N. cyanipes* have neo-XX/neo-XY sex chromosomes. They derived from the fusion of the ancestral X with one of autosomes. The small fragment containing centromere could arise as the by-product of the fusion. We suppose that this new small chromosome could evolve into modern B_s_. The association of these B_s_ with the terminal region of a short arm of neo-X chromosome (arm derived from the ancestral X) in meiosis (Figure 1b) is an additional argument for this hypothesis.

### 4.2. From Dot-Like B_s_ to the Large Ones

Traditional view on B chromosome evolution suggests that insertions into dot-like B_s_ followed by DNA amplification lead to large B_s_ [6]. Data on the B_s_ in *Podisma* species were involved earlier in the discussion of mechanisms of B chromosome evolution [34]. The DNA amplification leads to enlargement of their C-positive regions significantly increasing the size of the B_s_. The DNA amplified in the B_s_ includes DNA fragments from ancestor chromosome and fragments inserted into B_s_ on various stages of their evolution. A_s_ a result, large C-positive B_s_ contain different types of repeats, such as satDNA, mobile elements, and rDNA [34,35,36].

The high mobility of rDNA was shown for many species and in many grasshopper species the regions of B_s_ appeared to be enriched with rDNA [5]. In *E. plorans* and *P. sapporensis* many C-positive regions of B_s_ are enriched with rDNA [34,37]. Most part of rDNA in B_s_ is non-functional [38]. Probably, the amplification of rDNA is frequently involved into formation of large C-positive regions of A_s_ and B_s_. These repeats can contribute to molecular composition of pericentric C-positive regions [28,39] and in formation of numerous additional C-positive arms of A_s_, for instance, in *P. sapporensis* [31] and *Eremopeza festiva* [40]. However, the amplification of rDNA does not always occur in C-positive B_s_ of grasshoppers. In other species involved in this study, the B_s_ did not contain rDNA. It is possible that the amplification of rDNA takes place in the B_s_ that initially contained these repeats or in species with highly mobile genetic elements containing DNA homologous rDNA.

SatDNA is another type of repetitive sequences mainly present in pericentomeric C-positive regions of A_s_ in many species including grasshoppers [11,13,37,41]. In grasshoppers, containing no B chromosome satDNA represents small portion of their genomes. In specimens of *Pyrgomorpha conica* without B_s_ in their karyotypes, high-throughput analysis of satDNA revealed 87 satDNA variants, representing 9.4% of its genome [42]. In the main genome of *Locusta migratoria*, satDNA comprise only 2.4% of genomic DNA [42]. In contrast to the main genome, C-positive B_s_ are enriched with satDNA. In studied B chromosome of *Locusta migratoria*, 65.2% of its DNA is consisted of this type of repeats [42]. Abundance of a certain type of satellite repeats can be altered by the DNA amplification. In the B chromosome of *L. migratoria* more than a half of DNA refer to one type of satellite repeat [42]. In *E. plorans*, B_s_ are significantly enriched with 180 bp satDNA repeat [43]. However, satellitome studies on grasshopper B_s_ are in their beginning stage and additional intensive studies are required for understanding of the role of satDNA in B chromosome evolution.

The third type of repeats that can be involved in B chromosome development are mobile elements. It was thought that genetically inert B_s_ could serve as the targets for mobile element insertions and their accumulation [6]. However, earlier it was shown that mariner-like elements are located mostly in euchromatic regions of A_s_ and C-negative regions of B_s_. They were undetected in the regions enriched with rDNA and tandem repeats and in heterochromatic parts of the B_s_ [35,36]. Furthermore, study of the B chromosome in *L. migratoria* showed that transposable elements (TE) comprise only 18% of its content. It is significantly lower than TE content in A_s_ [42]. Probably, the role of TE in growth of C-positive regions of B chromosomes is nonessential.

Besides the insertion of rDNA, satDNA, and mobile elements, other repeats could also contribute to the B chromosome development. We had expected that in some B_s_, amplification of telomeric repeats could also take place like in development of large C-positive regions of chromosomes in stick insects [44]. However, to date large C-positive regions of B_s_ enriched with telomeric repeats have not been observed. The only exception was a region in the B chromosome of *B. gebleri* (Figure 5). Small unique DNA fragments of the main genome could probably also be inserted into the B_s_ without forming C-negative region. This phenomenon was described in studies of some B_s_ in mammals [19,20,21].

The factors determining the frequency of the dot-like B chromosome arising and their further evolution are associated with the basic genome particularities: location of hotspots of chromosome rearrangements close to centromere; high mobility of some genetic elements; predisposition to DNA amplification. All these genome characteristics and processes might play a similar role in different species. For instance, hotspots of chromosome rearrangements close to pericentromeric regions are associated with the small SMC arising in humans [33,45], the mobility of DNA fragments leads to formation of duplicon clusters [46,47,48], insertions of DNA fragments and their amplification could lead to the development of additional C-positive regions [49].

### 4.3. The B_s_ with C-Negative Regions

Karyotyping of some grasshopper species revealed mostly C-negative B_s_ and B_s_ with C-negative regions. These B_s_ can be divided into at least three groups according to their morphology and hypothetical evolutionary stage: (*i*) mostly C-negative B_s_ (Figure 2, Figure 3 and Figure 4); (*ii*) the B_s_ containing large C-positive and C-negative region(s) (Figure 5, Figure 6, Figure 7, Figure 8, Figure 9 and Figure 10); (*iii*) mostly C-positive B_s_ and C-positive B_s_ with small C-negative region(s) (Figure 7, Figure 8, Figure 9 and Figure 10). We consider the transposition of large C-negative regions from A_s_ into the B_s_ on an advanced stage of their evolution being at least rare events and for explanation of the existence of B_s_ with large C-negative region(s) we looked for different mechanisms of such B chromosome development.

Cell functioning is regulated by complex interactions of multiple genes and gene dosage balance is crucial in this process. Usually aneuploidy has detrimental effects on the ontogenesis [50]. The example of a high frequency of aneuploidy of the large chromosome in free-living flatworm *Macrostomum lignano* turned out to be hidden polyploidy [51,52,53]. At least partial inactivation of the B_s_ containing C-negative regions is required to avoid genetic imbalance [9] otherwise they should be prone to negative selection. In mostly heterochromatic B_s_, genes in their small C-negative regions are probably inactivated due to their close proximity to heterochromatic blocks. Human small SMCs with small euchromatic regions also show no visible effect on phenotype of their carriers [54]. This suggests that such B_s_ can appear to be neutral or almost neutral elements for natural selection. But for the mostly C-negative B_s_ the problem of dosage balance should still exist. Earlier, the X and small autosomes were considered the most probable sources for B_s_. The extra X chromosome could be tolerated due to dosage compensation mechanisms while small autosomes could be tolerated due to the low number of genes [6,9]. However, we should take into account that in some grasshoppers a high level of polysomy of some autosomes was observed and these polysomic elements do not cause problems in meiosis exhibiting signs of heteropicnocity and chromosome inactivation [55]. This suggests that even extra copies of autosomes in grasshoppers can escape from negative selection. These data inspired us to come back to consideration of large and medium autosomes as ancestors of B_s_ in more detail.

We revealed and described B_s_ in *Aeropus sibiricus* that are probably on their early evolutionary stage. The morphology and C-banding patterns of these B_s_ were very similar to the chromosomes of the basic set (Figure 2). Similar B_s_ were observed in other population of *A. sibiricus* and even initially misinterpreted as aneuploidy [56]; however, later they were referred to as B_s_ [57,58]. In meiosis, they formed multivalents with each other and did not interfere with bivalents of A_s_*.* Other early evolutionary stages of B_s_ were observed in *Chorthipps jacobsoni* and *Chorthippus apricarius* (Figure 3 and Figure 4), however, these B_s_ are probably on a more advanced stage of their evolution. In *C. jacobsoni*, the B_s_ underwent rearrangements resulted in iso-B_s_. More complex reorganization (such as inversions, new C-positive region development, and other rearrangements) has occurred in evolution of the B_s_ in *C. apricarius*. Occurrence of the whole C-negative B_s_ are rare; however, C-negative morphotypes of B_s_ were also described in Greek, Turkish and Armenian populations of *E. plorans* [16,32] and in *Abracris flavolineatus* [59].

The B_s_ containing large C-positive and C-negative regions are probably on a more evolutionary advanced stage. The B_s_ of this type are more frequent. They were revealed among of B_s_ in *E. plorans* [32,60,61,62] (Figure 9), *P. sapporensis* [30] (Figure 7)*, Bryodema geblery* (Figure 5), *Myrmeleotettix maculatus* [63], *Xyleus discoideus angulatus* [22], and in *L. migratoria* [64] showing various morphotypes. Often these chromosomes contain a large C-positive region and smaller alternating C-positive and C-negative regions. The morphology of these chromosomes can be acrocentric or metacentric (i.e., isochromosomes) (Figure 7 and Figure 9).

Finally, the massive part of the advanced B_s_ are mostly C-positive B_s_ or C-positive B_s_ with small C-negative region(s). Many described B_s_ of this type look like completely or almost completely heterochromatic. This stage of B chromosome evolution is indistinguishable from large B_s_ evolved from dot-like B_s_ after amplification of their DNA. However, some of these entirely C-positive B_s_ could contain small euchromatic regions visible only in very stretched chromosomes. For example, the C-positive Ba1 in *E. plorans* contained small regions painted with euchromatic DNA probe (Figure 10a,d). The sequencing of microdissected libraries of mammalian B_s_ revealed DNA fragments homologous to a unique DNA located in different chromosomes of the basic set [19,20,21]. The question “are the C-negative regions of the B_s_ the remnants of large euchromatic regions earlier present in the B or are they the result of multiple insertions/duplications of a small euchromatic chromosome region?” remains open. Probably both types of B_s_ are present in natural populations. Furthermore, in some B_s_ small insertions of DNA fragments from euchromatic regions of A_s_ seem to be too small to form visible C-negative regions in these B_s_ [19,20].

### 4.4. DNA Content of C-Negative Regions in B_s_

Grasshopper genomes are the largest genomes among insects and are significantly enriched with repeats [65,66,67,68]. These repeats are differentially distributed among C-positive and C-negative regions. For instance, C-negative regions contain more mobile elements than C-positive ones [35,36]. This allowed generating a microdissected DNA probe from C-negative regions that paints only C-negative chromatin [31] even in different closely related species [29,34,69]. However, in B_s_ C-negative regions can be enriched with repeats that differ from dispersed repeats of C-negative regions of A_s_. The C-negative B7 in *P. sapporensis* is a perfect example of such B_s_ [31]. FISH with DNA probe generated from a C-negative region of A chromosome gave signal only in a distal region of the B7. The rest of its C-negative regions remained unpainted. DNA content of the proximal part of the B7 remains unknown. If it was formed with DNA amplification of C-negative region of the A chromosome, it should have usual interspersed repeats and should be painted with a DNA probe derived from the euchromatic region of A chromosome [70]. The loss of all interspersed repeats from these regions is unlikely. In addition, we cannot accept the B chromosome origin from interspecific hybridization of closely related species. Interspersed repeats of euchromatic regions in *Podisma* species show high homology [34]. A_s_ concerns the C-negative B_s_ in the Greek population of *E. plorans*, they showed enrichment for rDNA and 180 bp satDNA [16]. Maybe these B_s_ are on the early stages of repeat expansion and they are not able to convert C-negative regions to C-positive ones. In *E. plorans*, we revealed the mostly C-negative autosome containing numerous dispersed clusters of repeats homologous to DNA of the Ba4 (Figure 10c), which supports this hypothesis. These intercalary repeat clusters are too small to form visible C-positive blocks and their distribution was limited with the only autosome, S10.

The C-negative regions of different sizes in the B_s_ pose additional questions about the DNA content of B_s_ and transcriptional activity of these genes. There is only a little information about their transcriptional activity. Recently, ten genes were discovered in the B_s_ of *E. plorans.* The *CIP2A*, *GTPB6*, *KIF20A*, and *MTG1* were complete while the *CKAP2*, *CAP-G*, *HYI*, *MYCB2*, *SLIT,* and *TOP2A* were truncated. At least a half of these genes are transcriptionally active [71]. It should be noted that these genes are involved in cell division processes as well as the genes of B_s_ of vertebrates [19,20,21,72,73,74]. However, the exact location and flanking regions of these genes are mostly unknown. Their location in C-positive regions could not be excluded. We should note that DNA transcription in heterochromatic regions was shown in many species on different stages of individual development and in some other special situations [75,76]. The role and significance of transcription taking place in the B_s_ should be thoroughly studied in the future.

### 4.5. Mechanisms of C-Negative B Chromosome Evolution

Two forces driving B chromosome evolution can be generally described as the DNA amplification from one side and the degradation of some chromosome regions from another side. We suppose that in further development of C-negative B_s_, their degradation will be probably the main process, while in dot-like B_s_ we should expect the amplification of their DNA. The mechanisms of degradation may include generation and distribution of repeat clusters along the euchromatic arm of the precursor chromosome like the distribution of repeat clusters along the euchromatic arm of the S10 in *E. plorans* (Figure 10c). The expansion of the repeats cluster along the chromosome probably occurred through inversions, transferring the part of cluster along the chromosome arm. After transposition, amplification of its DNA resulted in growing the repeat cluster in size. We suggest at least two consequences of this process. Due to inversions, crossing over between original and rearranged autosomes should be suppressed and the frequency of meiotic abnormalities involving them should be increased leading to a higher frequency of aneuploidy on this chromosome. Additionally, transcription activity of genes located between heterochromatic blocks could be decreased, saving the specimens with trisomy from negative natural selection. Subsequent deletions of C-negative regions located between repeat clusters could decrease genetic imbalance on this part of the genome and lead to the B chromosome arising. Smaller size of the Ba4 can be explained by the advanced stages of elimination of the euchromatic regions located between these repeat clusters. The elimination of euchromatic regions may eventually lead to a fusion of repeat clusters into a continuous region composed of repetitive DNA, showing pattern of C-positive staining. Among the studied B_s_ there are chromosomes containing proximal or distal enlarged C-positive regions. Probably, the distribution of repeat clusters along the chromosome arm followed by the elimination of euchromatic regions can start from the proximal or distal part of the chromosome, leading to the formation of a visible C-positive block in a corresponded chromosome region. A strong support of this hypothesis was provided by finding of the neo-Y degradation in Pamphagidae grasshoppers leading morphotypes of the neo-Ys that are similar to morphotypes of some studied B_s_ [27,28,29].

### 4.6. Lesson from neo-Y Chromosomes in Pamphagidae Grasshoppers

Degradation of euchromatic part of ancestor chromosome played a crucial role in neo-Y chromosome evolution in Pamphagidae grasshoppers. In contrast to standard XX♀/X0♂ sex determination chromosome system the neo-sex chromosomes are rare for grasshoppers. However, the frequency of the fusion of the autosome with the X chromosome in Pamphagidae grasshoppers appeared to be unusually high. The neo-Y chromosome in these grasshoppers undergoes a process of intensive degradation [27,29,40,77,78,79]. Different stages of the neo-Y evolution can be observed in species of this family. In early stages, the neo-Ys are fully homologous to the arm of the neo-X derived from the autosome. In the next stage of its development, the neo-Ys are characterized with small interstitial C-positive regions accumulated in its proximal part. More advanced neo-Ys appear to be smaller due to the deletion of C-negative regions located between C-positive blocks. The fusion of the small interstitial C-blocks forms the large ones [27,29]. Dissemination of repeats followed by the development of repeat clusters and the deletion of the euchromatic regions located between them on further stages may be a universal mechanism of the chromosome degradation in grasshoppers that could be also involved in process of the B chromosome development.

### 4.7. Many Ways to the B_s_

We cannot discard any hypothetical mechanisms suggested earlier [5,6,10,13,71,80] and mentioned above. On the contrary, we suppose that many of them or even a combination of them can be involved in the appearance and further evolution of the B_s_. Involvement of different mechanisms depends on particularities of the basic genome. Their diversity results in the great variety of the B_s_ that differ in their morphology, size, and DNA content. Nevertheless, being different in the initial stage, they can probably converge on a certain stage of their evolution acquiring similar morphology. However, then B_s_ could pass through further rearrangements to the next stage of evolution. Besides insertions and DNA amplification, B_s_ are also prone to converting to biarmed chromosomes. Probably the break near centromeric region and incorrect DNA reparation can result in iso-B_s_. Then each arm undergoes independent evolution. A similar mechanism of iso-B chromosome development was suggested for the B chromosome evolution in the Korean field mouse, *Apodemus peninsula* [81].

### 4.8. The Maintenance of B_s_ in Natural Populations

The existence of positive or negative natural selection against the specimens with B_s_ remains an open question. A strong positive selection in favor of the B_s_ should increase the frequency of specimens with the B_s_ in populations and probably increase their number per specimens. Strong negative selection should lead to the loss of the B_s_. There are species without the B_s_ and the B_s_ are present in the majority of specimens [82]. However, in many species the specimens with B_s_ represent a minor part in most of populations. From our point of view, such maintenance of the B_s_ requires at least two factors. For example, we can suggest weak negative natural selection against the B_s_ on one hand, and on the other, the preferential B chromosome transfer in oocyte or more efficient spermatocytes with the B_s_. This drive will increase the probability of the B chromosome transfer to the next generation. We should also consider an alternative suggestion: positive natural selection for carriers of the B_s_ and decreased frequency of the B chromosome transfer to the next generation. Data on the genes present in the B_s_ of *E. plorans* and their transcriptional activity [71] stimulate intensive study, keeping in mind both suggestions. It is also possible that among different kinds of B_s_ (the B_s_ differ by their origin, DNA composition, influence on adaptiveness of their carriers, and being at different stages of their evolution) there are different modes of selection. For instance, the loss of euchromatic regions in B_s_ neutralize the mode of selection, but enlargement of their C-positive regions will cause negative selection. The development of B_s_ can lead to switching of the natural selection direction and converting the maintenance of the B_s_ in populations to a dynamic process providing the permanent wide diversity of the B_s_ that depends on particularities of basic genome and habitat versatility.

## 5. Conclusion

The possible mechanisms of the appearance and evolution of the Bs in grasshoppers were analyzed and discussed. We suggest that different mechanisms can be involved in these processes. One of them includes the stage of a small supernumerary chromosome with the following stages of DNA fragment incorporation and amplification. Another mechanism could be associated with the distribution of repeat clusters along the arm of the A chromosome and the loss of euchromatic regions located between them. Both autosomes and sex chromosomes can be involved in the B chromosome’s appearance. It is probable that at least in some cases the process of the B chromosome evolution is similar to the neo-Y degradation described in Pamphagidae grasshoppers. The origination and evolution of the Bs depends on such particularities of the basic genome as a location of hotspots of chromosome rearrangements, mobility of various genomic elements, and a predisposition of some DNA fragments to their amplification. In some grasshoppers, rDNA or DNA fragments homologous to rDNA are prone to transpositions and amplification, leading in some species to enrichment of the B chromosome regions for DNA at least partly homologous to rDNA. The question of natural selection in favor or against the Bs is still open. Furthermore, we cannot rule out the switchover to different direction of natural selection during B chromosome evolution.

Further studies on the grasshopper Bs shall be devoted to identification of their euchromatic regions, and determination of their gene content and transcription activity, including its role and significance. The question that requires special attention concerns the determination of DNA content of mysterious C-negative B chromosome regions containing no or few repeats that are typical for euchromatic regions of As.

## Figures and Tables

**Table 1:** Studied species and locations of their collection.

Taxa	Species Name	Location	Year	*N*
Acrididae				
Gomphocerinae	*Chorthippus apricarius* (L.)	Foothills of Trans-Ili Alatau mountains near Almaty, Kazakhstan	2005	3 ʘ from 2♀
	*Chorthippus jacobsoni* (Ikonn.)	Foothills of Trans-Ili Alatau mountains near Almaty, Kazakhstan	2007	7 ʘ from 3♀
	*Aeropus sibiricus* (L.)	Kurai steppe, Altay mountains, Russian Federation	2007	6♂
Oedipodinae	*Bryodema gebleri* (F-W.)	Kurai steppe, Altay mountains, Russian Federation	2017	12♂
Melanoplinae	*Podisma sapporensis sapporensis* Shir.	Hokkaido, Japan	2000, 2005	157♂, 37 ʘ
Eyprepocnemidinae	*Eyprepocnemis plorans plorans* (Charp.)	Megri and Yerevan, Armenia, Antalia, Turkey, Malaga, Spain (kindly provided by JPM Camacho)	2003, 2007	381♂ 551♂
	*Eyprepocnemis plorans meridionalis* Uv.	Springbok, South Africa	2004	33♂
**Pamphagidae**				
Pamphaginae	*Nocaracris tardus* Ünal, Bugrov & Jetybayev	Sultandag mountains, Turkey	2014	7♂
	*Nocaracris cyanipes* (F-W.)	Prielbrusie National park Kabardino-Balkaria, Russia	1987	5♂
Trinchinae	*Asiotmethis heptapotamicus songoricus* Shum.	Ayagoz, East Kazakhstan	2015	18♂

*N*—Number of studied samples; ʘ—eggpods.

**Table 2:** Microdissected DNA probes.

DNA probe	Species	Dissected Chromosome/Region
WCPNtaB	*Nocaracris tardus*	Whole B
PCPCapBq	*Chorthippus apricarius*	Long arm of the B
PCPCapAc	*Chorthippus apricarius*	Pericentric region of autosome
WCPEplBa4	*Eyprepocnemis plorans*	Whole Ba4
PCPPsaB1-B2dist	*Podisma sapporensis*	Distal parts of B1 and B2
